# An Extract of *Rhodobacter sphaeroides* Reduces Cisplatin-Induced Nephrotoxicity in Mice

**DOI:** 10.3390/toxins5122353

**Published:** 2013-11-29

**Authors:** Wen-Wei Chang, Jau-Jin Liu, Chi-Fan Liu, Wen-Sheng Liu, Yun-Ping Lim, Yu-Jung Cheng, Che-Hsin Lee

**Affiliations:** 1 Department of Biomedical Sciences, College of Medical Science and Technology, Chung Shan Medical University, Taichung 402, Taiwan; E-Mail: changww@csmu.edu.tw; 2 Department of Medical Research, Chung Shan Medical University Hospital, Taichung 402, Taiwan; 3 Department of Microbiology, School of Medicine, China Medical University, Taichung 404, Taiwan; E-Mails: jjl@mail.cmu.edu.tw (J.-J.L.); ruz0417@yahoo.com.tw (C.-F.L.); 4 Asia-Pacific Biotech Developing, Inc. Kaohsiung 806, Taiwan; E-Mail: wensheng5394@gmail.com; 5 Department of Marine Biotechnology and Resources, National Sun Yat-sen University, Kaohsiung 804, Taiwan; 6 Department of Pharmacy, College of Pharmacy, China Medical University, Taichung 404, Taiwan; E-Mail:limyp@mail.cmu.edu.tw; 7 Department of Physical Therapy, Graduate Institute of Rehabilitation Science, China Medical University, Taichung 404, Taiwan; E-Mail: chengyu@mail.cmu.edu.tw

**Keywords:** *Rhodobacter sphaeroides*, Lycogen™, cisplatin, nephrotoxicity

## Abstract

Cisplatin is used as a treatment for various types of solid tumors. Renal injury severely limits the use of cisplatin. Renal cell apoptosis, oxidative stress, and inflammation contribute to cisplatin-induced nephrotoxicity. Previously, we found that an extract of *Rhodobacter sphaeroides* (Lycogen™) inhibited proinflammatory cytokines and the production of nitric oxide in activated macrophages in a dextran sodium sulfate (DSS)-induced colitis model. Here, we evaluated the effect of Lycogen™, a potent anti-inflammatory agent, in mice with cisplatin-induced renal injury. We found that attenuated renal injury correlated with decreased apoptosis due to a reduction in caspase-3 expression in renal cells. Oral administration of Lycogen™ significantly reduced the expression of tumor necrosis factor-α and interleukin-1β in mice with renal injury. Lycogen™ reduces renal dysfunction in mice with cisplatin-induced renal injury. The protective effects of the treatment included blockage of the cisplatin-induced elevation in serum urea nitrogen and creatinine. Meanwhile, Lycogen™ attenuated body weight loss and significantly prolonged the survival of mice with renal injury. We propose that Lycogen™ exerts anti-inflammatory activities that represent a promising strategy for the treatment of cisplatin-induced renal injury.

## 1. Introduction

Cisplatin, a platinum anticancer drug, has been widely used for the treatment of malignant tumors. The important dose-limiting factor of cisplatin is nephrotoxicity. Renal cell apoptosis, oxidative stress, and inflammation have been recognized as the mechanisms for cisplatin-induced nephrotoxicity [1].

It was proposed that bacteria may reduce inflammation by controlling the production of proinflammatory cytokines [2,3]. Bacteriopurpurinimides, derived from *Rhodobacter sphaeroides*, are highly stable and potent photosensitizers used in photodynamic therapy [4]. *R. sphaeroides* contains carotenoids that have anti-inflammation activity [5,6]. Therefore, therapeutic strategies utilizing *R. sphaeroides* extracts may decrease inflammation and protect renal injury after cisplatin treatment. Lycogen™ is an extract of *R. sphaeroides* that displays anti-inflammatory and anti-oxidative abilities [7]. These observations raised the interesting possibility that Lycogen™ may be responsible for inhibiting cytokine production and renal dysfunction during cisplatin-induced renal injury.

## 2. Results

### 2.1. Evaluation of Cell Viability after Lycogen™ or Cisplatin Treatment *in Vitro*

**Figure 1:**
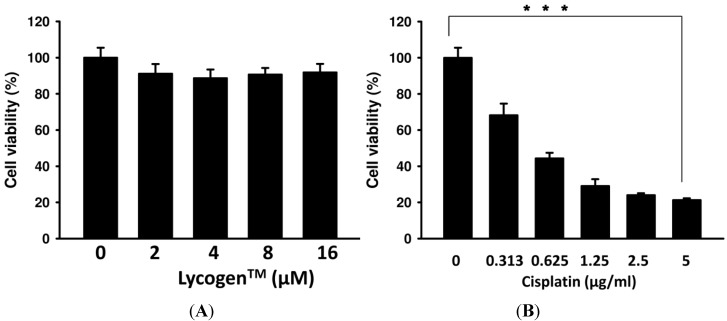
The effects of Lycogen™ and cisplatin on the viability of mesangial cells (MES-13). MES-13 cells were treated with the indicated concentrations of Lycogen™ or cisplatin for 48 h. Cell viability was measured after (**A**) Lycogen™ treatment or (**B**) cisplatin treatment using a WST-1 assay. *** *p* < 0.001 (mean ± SD, *n* = 6). Each experiment was repeated three times with similar results.

In this study, we used mouse glomerular mesangial cells (MES-13) to evaluate the potential cytotoxic effect of Lycogen™. Previously, we used a 48-h treatment of Lycogen™ as an anti-melanogenic agent in cells [8]. In this study, we again chose this time course to measure the preventive effect of Lycogen™ on cisplatin-induced cell death. As shown in Figure 1, Lycogen™ did not significantly alter cell survival at doses ranging from 2 μM to 16 μM (Figure 1A). The cell viability of MES-13 cells dramatically decreased after treatment with cisplatin (Figure 1B). These results suggest that Lycogen™ shows no cytotoxic effect on MES-13 cells when compared with cisplatin-treated cells.

### 2.2. Lycogen™ Treatment Attenuated Cisplatin-Induced Renal Cell Death

**Figure 2:**
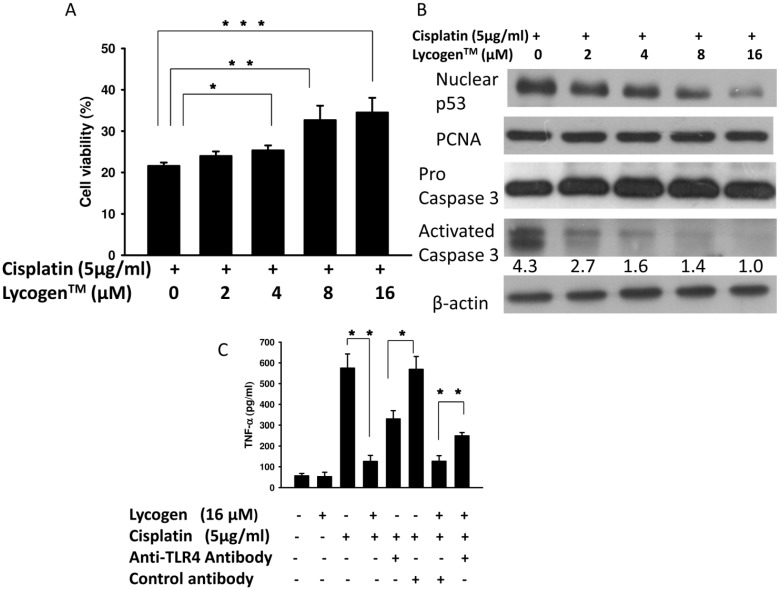
Lycogen™ reduced cisplatin-induced cell apoptosis. MES-13 cells were pretreated with Lycogen™ at concentration of 0, 2, 4, 8 or 16 μM for 48 h. Next, cisplatin (5 μg/mL) was added to the cells for 48 h. (**A**) Cell viability was measured using a WST-1 assay; (**B**) The expression of nuclear p53 and caspase 3 was measured by Western blot analysis. Proliferating cell nuclear antigen (PCNA) and β-actin expression served as loading controls for nuclear proteins and total proteins, respectively. Inserted values indicate the relative protein expression when compared with pro-caspase 3. * *p* < 0.05, ** *p* < 0.01, *** *p* < 0.001 (mean ± SD, *n* = 6); (**C**) TNF-α secreted by MES-13 cells is mediated by TLR4 signaling. The cells were preincubated with anti-TLR4 or with control IgG, and then treated with or without Lycogen™ for 48 h. TNF-α was measured using an enzyme-linked immunosorbent assay. Each experiment was repeated three times with similar results.

We evaluated the effect of Lycogen™ and cisplatin on MES-13 cells and found that MES-13 cells pretreated with Lycogen™ (16 μM) showed a significant increase in cell survival after cisplatin treatment (34.50% ± 3.56% *versus* 21.62% ± 0.80%) (Figure 2B). Lycogen™ reduced cisplatin-induced cell death. Cisplatin-induced caspase 3 activation is not mediated by decreases in cellular energetics or mitochondrial membrane potential because it is independent of caspase 8 or 9 in renal cells [9]. Furthermore, p53 functions upstream of caspase 3 and mediates its activation during cisplatin-induced renal cell apoptosis [9]. In this study, cisplatin-treated MES-13 cells showed increased activation of caspase 3 and nuclear p53 (Figure 2B). However, treatment with Lycogen™ dose-dependently reduced the expression of caspase 3 and the accumulation of nuclear p53 in MES-13 cells. TNF-α plays an important role in the pathogenesis of cisplatin-induced renal injury. Cisplatin stimulates TNF-α production in the kidney through the mitogen-activated protein kinase (MAPK) signaling pathway [10]. Toll-like receptor (TLR) 4 also contributes to the activation of TNF-α in kidney injury. TLR4 deficiency is associated with reduced kidney injury and an attenuated proinflammatory state [11]. Although kidney cells express or release factors that are potent TLR4 activators after cisplatin treatment, certain endogenous ligands have been found to interact with TLR4. The lipopolysaccharide (LPS) of *R. sphaeroides* is an effective TLR4 antagonist [12,13]. Thus, it is important to explore the hypothesis that TLR4 function contributes to TNF-α production in cisplatin-treated renal cells. We found that the level of TNF-α, a proinflammatory cytokine, was elevated in the cisplatin treated group when compared with the control. Pretreatment of MES-13 cells with Lycogen™ decreased the level of TNF-α (Figure 2C). Next, we sought to determine if TNF-α was present in the TLR4-dependent soluble mediators secreted by MES-13 cells. The level of TNF-α was significantly decreased in cisplatin-treated MES-13 cells treated with a neutralizing TLR4 antibody, but not in those treated with control antibodies (Figure 2C). These results suggest that TLR4 plays a role in the activation of TNF-α in cisplatin-induced kidney injury. As shown in Figure 2C, the level of TNF-α in MES-13 cells pre-treated with neutralizing TLR4 antibodies and Lycogen™ was increased when compared with the group treated with control antibodies and Lycogen™ (249.41 ± 16.24 pg/mL *versus* 126.47 ± 27.17 pg/mL, *p* = 0.0025). Therefore, Lycogen™ may partially inhibit TLR4 signaling and reduce TNF-α expression after cisplatin treatment in MES-13 cells. Our results demonstrate that Lycogen™ treatment markedly attenuated cisplatin-induced renal cell death.

### 2.3. Lycogen™ Treatment Attenuated Renal Injury in Cisplatin-Treated Mice

**Figure 3:**
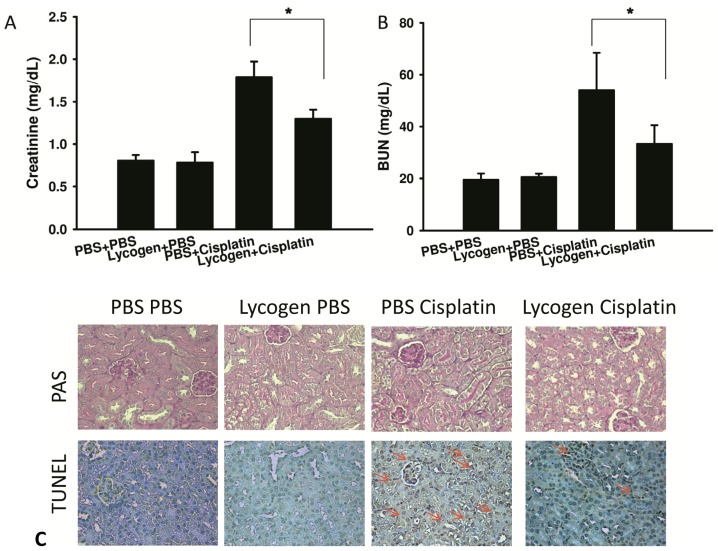
Lycogen™ ameliorated cisplatin-induced renal dysfunction. Mice were treated with Lycogen™ (1 mg/kg) for three consecutive days, starting on day 0. Mice were given an intraperitoneal injection (i.p.) of cisplatin (30 mg/kg) on day 3. Control mice received PBS. The effect of Lycogen™ on (**A**) creatinine and (**B**) blood urine nitrogen (BUN) levels 72 h after cisplatin administration. * *p* < 0.05 (mean ± SD, *n* = 4). (**C**) Sections of kidney were stained with PAS or TUNEL 72 h after the cisplatin injection. The arrows indicate the location of positive TUNEL staining in the kidney. Each experiment was repeated three times with similar results.

**Figure 4:**
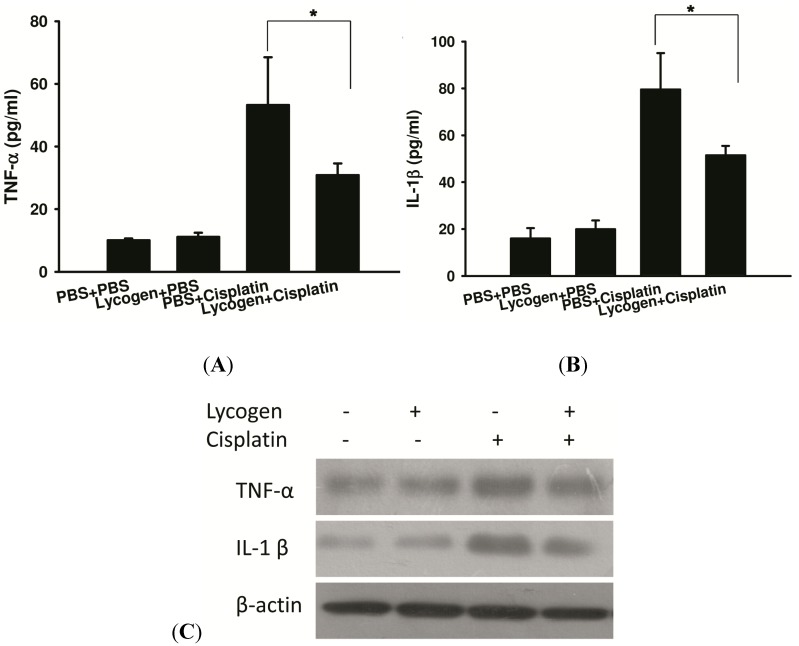
Lycogen™ ameliorated cisplatin-induced renal inflammation. Mice were treated with Lycogen™ (1 mg/kg) for three consecutive days starting on day 0. Next, mice were injected i.p. with cisplatin (30 mg/kg) on day 3. Control mice received PBS. (**A**) TNF-α and (**B**) IL-1β levels in the sera as measured by ELISA 72 h after cisplatin treatment. * *p* < 0.05 (mean ± SD, *n* = 4). (**C**) Lycogen™ reduced cytokine expression in the kidney. A portion of the mice received Lycogen™ treatment. After three days, mice were killed, kidneys were collected, and the renal lysates were analyzed for TNF-α and IL-1β expression using immunoblot analysis. Each experiment was repeated three times with similar results.

Renal function was monitored using serum creatinine and blood urea nitrogen (BUN). Cisplatin administration caused a 2-fold and 3-fold increase in creatinine and BUN, respectively, *in vivo*. Treatment with Lycogen™ led to a 27% reduction in serum creatinine and 38% reduction in BUN compared cisplatin treated mice. No significant differences were observed in creatinine and BUN between mice treated with PBS and Lycogen™ (Figure 3A,B). To investigate the effect of Lycogen™ on cisplatin-induced renal injury, we stained kidney sections with periodic acid Schiff (PAS) and used a terminal dUTP nick-end labeling (TUNEL) assay. Accumulation of PAS positive materials in the lumen and atrophy and degeneration of epithelial cells was observed in the cisplatin-treated group. The mice pretreated with Lycogen™ showed reduced cisplatin-induced histological damage. We measured the number of apoptotic cells within renal areas using a TUNEL assay. Lycogen™ significantly decreased cisplatin-induced apoptosis in the kidney (Figure 3C). To evaluate the effect of Lycogen™ treatment on cisplatin-induced inflammation, we measured the protein expression of the proinflammatory cytokines tumor necrosis factor-α (TNF-α) and interleukin-1β (IL-1β) in the serum. As shown in Figure 4, cisplatin induced the protein expression of IL-1β and TNF-α; furthermore, Lycogen™ suppressed TNF-α expression by 45% (66.86 ± 9.37 pg/mL *versus* 36.70 ± 9.00 pg/mL) and IL-1β expression by 55% (76.19 ± 21.57 pg/mL *versus* 34.02 ± 5.42 pg/mL). The expression of inflammatory cytokines was also determined in renal tissues using immunoblot analysis. These results were similar to the cytokine expression levels found in the sera. Taken together, these data show that oral administration of Lycogen™ can reduce the production of proinflammatory cytokines in a model of cisplatin-induced renal inflammation.

### 2.4. Lycogen™ Improved Cisplatin-Induced Body Weight Loss and Survival

**Figure 5:**
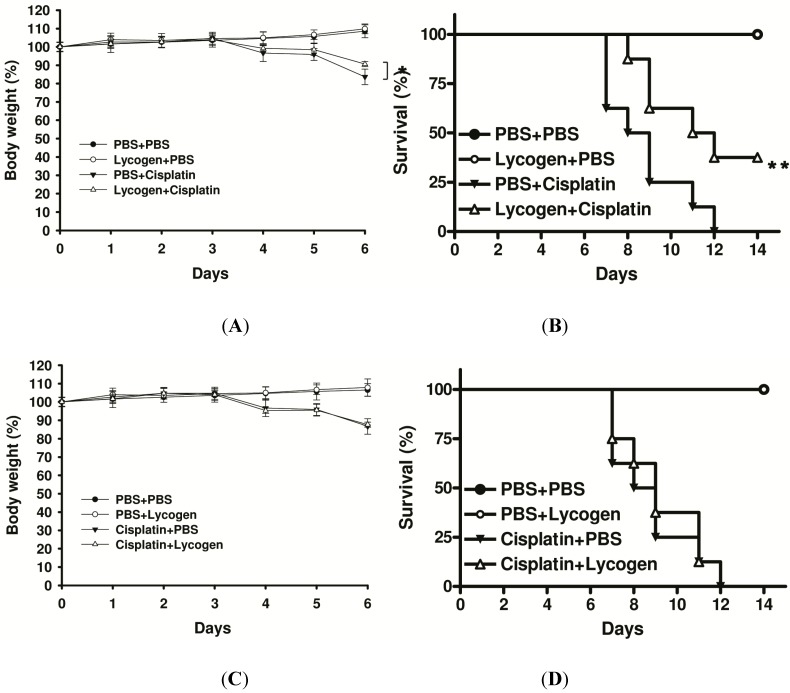
The effect of Lycogen™ on cisplatin-induced renal injury in mice. (**A**) The mice were orally administered Lycogen™ (1 mg/kg) for three consecutive days after an i.p. injection of cisplatin (30 mg/kg) and the body weight of mice was recorded daily (mean ± SD, *n* = 8, * *p* < 0.01 for cisplatin-induced renal injury mice pretreated with Lycogen™ *versus* cisplatin-induced renal injury mice pretreated with PBS); (**B**) Kaplan-Meier survival curves up to day 14 are shown (*n* = 8). Significant differences between continuous variables was assessed using Student’s t-test. Mice survival analysis was performed using the Kaplan-Meier survival curve and log-rank test. * *p* < 0.05, ** *p* < 0.01; (**C**) The mice were injected i.p. with cisplatin (30 mg/kg) and then orally administered Lycogen™ (1 mg/kg) for three consecutive days beginning 16 h after the cisplatin administration. The body weights of mice were recorded (mean ± SD, *n* = 8); (**D**) Kaplan-Meier survival curves up to day 14 are shown (*n* = 8).

We assessed multiple symptomatic renal failure parameters, including body weight and survival, caused by injury after cisplatin (30 mg/kg) administration (Figure 5). The mice treated with Lycogen™ showed a significant reduction in the loss of body weight induced by cisplatin administration (Figure 5A). Oral treatment with Lycogen™ alone did not influence the body weight and survival of mice, suggesting that Lycogen™ is safe for mice. Furthermore, Lycogen™ dramatically prolonged the survival of mice with severe renal injury (Figure 5B). The reported preventive effects of Lycogen™ on kidney injury models was observed. Using a therapeutic experimental design, Lycogen™ was administered 16 h after cisplatin injection. No changes in loss of body weight or survival were found after the therapeutic Lycogen™ treatment (Figure 5C,D). Taken together, these results suggest that Lycogen™ can have protective effects in cisplatin-induced renal injury.

## 3. Discussion

Cisplatin is a commonly used chemotherapeutic drug. The major limiting factor in the use of cisplatin is the side effects in normal tissues, including the kidney. Renoprotective approaches have been discovered; however, the effects are limited. Our current study demonstrates that a bacteria-derived molecule can improve epithelial cell injury. In this study, we found that mice fed diets containing Lycogen™ (1 mg/kg) for 3 days had no effect on body weight and proinflammatory cytokine production. Moreover, no adverse side effects were observed in mice that consumed the Lycogen™ diet (Figure 5). At the tested concentrations, no significant toxicity of Lycogen™ was observed in mice. 

Cisplatin induced nephrotoxicity occurs through oxidative stress, renal cell apoptosis, inflammation and vascular factors. Oxidative stress has been observed in patients with nephrotoxicity. These findings imply that the anti-oxidative potential of Lycogen™ may have the ability to inhibit cisplatin-induced renal injury. Inflammatory cytokines play an important role in the pathogenesis of nephrotoxicity. High levels of the pro-inflammatory cytokines IL-1β and TNF-α are present in patients suffering from nephrotoxicity. There is growing recognition of the importance of inflammation in the pathogenesis of cisplatin-induced nephrotoxicity. Cisplatin administration caused a 4-fold and 8-fold increase in TNF-α and IL-1β, respectively. Treatment with Lycogen™ led to a 45% reduction in TNF-α and a 55% reduction in IL-1β. Our results showed that Lycogen™ reduces functional and histological renal damage caused by cisplatin.

Use of a pan-caspase inhibitor or p53 inhibitor prevented cisplatin-induced renal tubular epithelial cell death, suggesting that caspases and p53 are involved in the response to cisplatin injury [14]. Previous studies have reported p53-dependent transcriptional control of the caspase genes and its functional significance in cisplatin-induced renal injury [15]. In addition, the induction of mitogen-activated protein kinase activation in oxidant injury contributes to the regulation of caspase activation [16]. In this study, we also found a marked increase in cisplatin-induced caspase 3 and nuclear p53 in MES-13 cells.

The LPS of *Rhodobacter* inhibited TNF-α induction in activated mononuclear cells [17]. It has been suggested that the LPS of *Rhodobacter* inhibited the production of cytokines on cells by at least two mechanisms, including blocking LPS receptor recognition and depletion of the cofactor LPS-binding protein [18,19]. Lycogen™ (an extract of *R. sphaeroides*) might contribute to the reduction of the immune response in cisplatin-induced kidney injury by inhibiting TLR4 signaling. Moreover, TLR4 signaling increases proinflammatory cytokine production and renal injury in experimental models. Our data also show that inhibition of TLR4 signaling in renal cells decreases the secretion of proinflammatory cytokines (Figure 2C). Lycogen™ may partially inhibit TLR4 signaling and reduce TNF-α expression after cisplatin treatment in MES-13 cells. Other factors involved in the protective role of Lycogen™ in cisplatin-induced renal injury, such as oxidative stress, require further investigation. 

Lycogen™ contains ζ-carotene, neurosporene, spheroidenone and methoxyneurosporene according to nuclear magnetic resonance spectroscopy analysis. ζ-Carotene is the precursor of neurosporene, which in turn is the precursor of lycopene [20]. Lycopene is a potent antioxidant and quencher of singlet oxygen [21]. These findings suggest that the anti-oxidative and anti-inflammatory potential of Lycogen™ might contribute to its protective effect on cisplatin-induced renal cell death (Figure 2B). In conclusion, our work has identified Lycogen™ as an anti-inflammatory agent with the capacity to ameliorate cisplatin-induced nephrotoxicity. However, further work is needed to elucidate the underlying mechanism behind the therapeutic effects of Lycogen™ on cisplatin-induced nephrotoxicity. 

## 4. Experimental Section

### 4.1. Reagents, Cells and Mice

*R.* *sphaeroides* (WL-APD911) was isolated from mutants using chemical mutagenesis (Bioresource Collection and Research (BCRC), Hsinchu, Taiwan). The *R. sphaeroides* was cultured in broth. After harvesting, the bacterial broth was centrifuged and washed with ethanol. The bacterial residue was extracted with acetone and then centrifuged at 7500 rpm for 5 min. The supernatant was filtered through filter paper and a 0.2 μm filter into a round-bottomed flask. The color of the final supernatant was dark red. Acetone was removed completely using an oven set at 55 °C. The *R. sphaeroides* extract was named Lycogen™ [7]. Lycogen™ is available from Asia-Pacific Biotech Developing, Inc. (Kaohsiung, Taiwan). Lycogen™ was dissolved in PBS. The MES-13 cell line (glomerular mesangial cells from an SV40 transgenic mouse) was obtained from American Type Culture Collection (CRL-1927; Manassas, VA, USA) and maintained in culture medium with a 3:1 mixture of DMEM and Ham’s medium, supplemented with 14 mM HEPES, 2 mM glutamine, antibiotics (100 μg/mL penicillin and 100 μg/mL streptomycin) and 5% FBS at 37 °C. The incubation chamber was equilibrated with 5% CO_2_-95% air. Male C57BL/6 mice were obtained from the Taiwan National Laboratory Animal Center. The experimental protocol adhered to the rules of the Animal Protection Act of Taiwan. The experimental protocol was approved by the Laboratory Animal Care and Use Committee. All mice were kept under standard conditions of temperature and light, and were fed with standard laboratory chow and water. Cisplatin was purchased from Sigma-Aldrich (St. Louis, MO, USA).

### 4.2. Cell Proliferation Assay

Cells (10^5^/well) were treated with various concentrations of Lycogen™ or cisplatin in the culture medium. Next, the medium was removed, and cells were washed and replenished with fresh medium supplemented with 2% FBS. Cell proliferation was assessed using the colorimetric WST-1 assay (Dojindo Labs, Tokyo, Japan) according to the manufacturer’s instructions [22].

### 4.3. Western Blot Analysis

Cell lysates were prepared by extracting proteins with lysis buffer. Cytoplasmic and nuclear fractions were prepared according to the manufacturer’s instructions (Pierce Biotechnology, Rockford, IL, USA). Proteins from total cell extracts were fractionated using SDS-PAGE, transferred onto Hybond enhanced chemiluminescence nitrocellulose membranes (Amersham, Little Chalfont, UK), and probed with primary antibodies against caspase 3 (GeneTex, San Antonio, TX, USA), p53 (Santa Cruz Biotechnology, Santa Cruz, CA, USA), proliferating cell nuclear antigen (PCNA) (GeneTex, Irvine, CA, USA), TNF-α (Santa Cruz Biotechnology, Santa Cruz, CA, USA), IL-1β (Santa Cruz Biotechnology, Santa Cruz, CA, USA) or monoclonal antibodies against β-actin (AC-15, Sigma-Aldrich). Horseradish peroxidase-conjugated secondary antibodies were used, and protein-antibody complexes were visualized with an enhanced chemiluminescence system (GE Healthcare Bio-Sciences, Pittsburgh, PA, USA). The signals were quantified with ImageJ software [23,24].

### 4.4. Establishment of Experimental Renal Injury Model

Male C57BL/6 mice at 6–8 weeks of age were given an intraperitoneal (i.p.) injection of cisplatin (30 mg/kg) as previously described [25,26]. Previously, the mice with colitis were orally administered with Lycogen™ (1 mg/kg) for six consecutive days after DSS induction and the body weights of mice were recorded. We found that this dose of Lycogen™ did not influence the body weight or survival of mice [7]. Therefore, 1 mg/kg of Lycogen™ was used throughout the experiments in the current study. To study preventive effects, Lycogen™ (1 mg/kg/day) was orally administered for three consecutive days by gavage. Control mice were treated with PBS. Mice were injected i.p. with cisplatin on day 3. To study therapeutic effects, mice were injected i.p. with cisplatin at day 0. Next, Lycogen™ was given 16 h after cisplatin administration for three consecutive days. Body weight loss was calculated as the percent difference between the original body weight and the body weight on each subsequent day.

### 4.5. Determination of Creatinine and BUN

Groups of mice were orally administered Lycogen™ (1 mg/kg) for three consecutive days by gavage, and then mice were injected i.p. with cisplatin (30 mg/kg) on day 3. Renal function was assessed by determination of creatinine and BUN using a creatinine assay kit (BioVision, Milpitas, CA, USA) and urea assay kit, respectively, (BioVision, Milpitas, CA, USA) on day 6.

### 4.6. Assessment of Cytokines

To determine the expression of TNF-α and IL-1β, mice were treated with Lycogen™ (1 mg/kg) for three consecutive days by oral gavage, and then mice were injected i.p. with cisplatin (30 mg/kg) on day 3. To detect cytokine expression, blood serum was collected on day 6. Cytokine levels in the sera were determined by ELISA (R&D, Minneapolis, MN, USA) [27,28]. The MES-13 cells were preincubated with 10 μg of neutralizing antibody against mouse TLR4 (MTS510, eBioscience, San Diego, CA, USA) or with control IgG (eBioscience) for 30 min at 25 °C, and then treated with Lycogen™ or cisplatin in the culture medium for 48 h. The level of TNF-α in the supernatant was determined by ELISA (R&D).

### 4.7. Histological Examinations

The mouse kidneys were sectioned in blocks, fixed in 4% paraformaldehyde, and then embedded in paraffin. The kidney block was cut into 8-μm thick sections and stained with PAS reagents [26]. The TUNEL assay was used to detect cell apoptosis in renal areas and was performed according to the manufacturer’s protocol (Promega, Madison, WI, USA). TUNEL-positive cells (brown staining) were quantified using microscopy [29].

### 4.8. Statistical Analysis

All data are expressed as the mean ± standard deviation (SD). An unpaired, two-tailed Student’s t-test was used to determine differences between groups. The mice survival analysis was performed using the Kaplan-Meier survival curve and log-rank test. A *p* value less than 0.05 was considered statistically significant.

## 5. Conclusions

In conclusion, our work has identified Lycogen™ as an anti-inflammatory agent with the capacity to ameliorate cisplatin-induced nephrotoxicity. However, further work is warranted to elucidate the underlying mechanism behind the protective effects of Lycogen™ therapy in cisplatin-induced nephrotoxicity.
